# Dose Optimization of Vancomycin for Critically Ill Patients Undergoing CVVH: A Prospective Population PK/PD Analysis

**DOI:** 10.3390/antibiotics10111392

**Published:** 2021-11-13

**Authors:** Chuhui Wang, Chao Zhang, Xiaoxiao Li, Sixuan Zhao, Na He, Suodi Zhai, Qinggang Ge

**Affiliations:** 1 Department of Pharmacy, Peking University Third Hospital, Beijing 100191, China; chuhui_wang@163.com (C.W.); laural.zhang@yahoo.com (C.Z.); lxxsunshine@sina.com (X.L.); qiuxuaner@126.com (S.Z.); hn12141122@163.com (N.H.); 2 Department of Pharmacy Administration and Clinical Pharmacy, School of Pharmaceutical Science, Peking University, Beijing 100191, China; 3 Department of Pharmacy, Beijing Tongren Hospital, Capital Medical University, Beijing 100730, China; 4 Critical Care Medicine Department, Peking University Third Hospital, Beijing 100191, China

**Keywords:** vancomycin, CVVH, dose optimization, population pharmacokinetics

## Abstract

The optimal dose of vancomycin in critically ill patients receiving continuous venovenous hemofiltration (CVVH) remains unclear. The objective of this study was to identify factors that significantly affect pharmacokinetic profiles and to further investigate the optimal dosage regimens for critically ill patients undergoing CVVH based on population pharmacokinetics and pharmacodynamic analysis. A prospective population pharmacokinetic analysis was performed at the surgical intensive care unit in a level A tertiary hospital. We included 11 critically ill patients undergoing CVVH and receiving intravenous vancomycin. Serial blood samples were collected from each patient, with a total of 131 vancomycin concentrations analyzed. Nonlinear mixed effects models were developed using NONMEM software. Monte Carlo Simulation was used to optimize vancomycin dosage regimens. A two-compartment model with first-order elimination was sufficient to characterize vancomycin pharmacokinetics for CVVH patients. The population typical vancomycin clearance (CL) was 1.15 L/h and the central volume of distribution was 16.9 L. CL was significantly correlated with ultrafiltration rate (UFR) and albumin level. For patients with normal albumin and UFR between 20 and 35 mL/kg/h, the recommended dosage regimen was 10 mg/kg qd. When UFR was between 35 and 40 mL/kg/h, the recommended dosage regimen was 5 mg/kg q8h. For patients with hypoalbuminemia and UFR between 20 and 25 mL/kg/h, the recommended dosage regimen was 5 mg/kg q8h. When UFR was between 25 and 40 mL/kg/h, the recommended dosage regimen was 10 mg/kg q12h. We recommend clinicians choosing the optimal initial vancomycin dosage regimens for critically ill patients undergoing CVVH based on these two covariates.

## 1. Introduction

Sepsis is a life-threatening organ dysfunction due to a dysregulated host response to infection [1,2]. Sepsis and the subsequent inflammation can lead to multiple organ dysfunction syndrome (MODS) and death [3]. Patients with MODS often need multiple life support treatments, such as continuous renal replacement therapy (CRRT) [4,5]. CRRT consists of continuous venovenous hemofiltration (CVVH), continuous venovenous hemodialysis (CVVHD), and continuous venovenous hemodiafiltration (CVVHDF). CVVH is a widely used mode in clinical practice [6], which can effectively remove most of the metabolites, as well as inflammatory mediators, toxins and other macromolecular substances. Meanwhile, CVVH can also remove some drugs by convection and ultrafiltration at the same time.

For sepsis patients, complicating factors can increase the prevalence of multidrug resistant infections, including methicillin-resistant *Staphylococcus aureus* (MRSA) [7,8]. Vancomycin is the first-line treatment of severe infection caused by MRSA [9], and it was often used as the treatment of sensitive bacteria or the empirical treatment of serious positive *coccal* infection. The molecular weight of vancomycin is 1448 Da, the plasma protein binding rate is around 55% (15–75%), and the apparent volume of distribution is about 0.7 L/kg (0.3–0.9 L/kg) [10]. Therefore, part of free drugs may be filtered out. A previous study showed that approximately one-fifth (213 mg) of vancomycin can be cleared after 12 h of CVVH when 1000 mg vancomycin was administered [11].

To maximize patients’ survival, timely and optimal administration of antibiotics is vital [4,12,13]. Therapeutic antibiotic concentrations should be attained rapidly and maintained diligently in order to maximize efficacy [4,14,15,16] and minimize toxicity [17,18,19]. However, the optimal dose of vancomycin in critically ill patients receiving CVVH remains unclear. Several studies [11,20,21,22,23] have investigated the topic, but their recommendations are controversial. Besides, the covariates of these studies were not fully considered, and most studies only recommended dosage regimens of one subgroup, which restricts the applicability to other patients. At present, the clinical dosage regimen for this population is mostly based on doctors’ experience, without clear evidence. Therefore, determining the dosage regimen of vancomycin for critically ill patients receiving CVVH is necessary. The aim of this study was to establish a population pharmacokinetic model and to quantitatively investigate vancomycin pharmacokinetic characteristics, variation and related influence factors of this special population. Besides, we aimed to determine the initial dosage regimen of vancomycin by model-based simulation.

## 2. Results

### 2.1. Patient Characteristics

A total of 131 vancomycin concentrations from 11 patients were included for analysis. Patient demographic characteristics are summarized in Table 1. Most of the patients were older than 60 years. The Acute Physiology and Chronic Health Evaluation II (APACHE II) scores mainly ranged from 20 to 25, and the Sequential Organ Failure Assessment (SOFA) scores mainly ranged from 8 to 10. Ten of eleven patients’ urinary output was less than 2000 mL. Three patients were anuria (urinary output < 100 mL) and three patients were oliguria (urinary output < 400 mL). Creatinine clearance before CVVH of nine patients was lower than 40 mL/min, which means the residual renal function was low of the included patients. Besides, as vancomycin is not metabolized in vivo and it is eliminated more than 90% in unchanged form, so complications and drug-drug interaction were not considered.

### 2.2. Model Development

#### 2.2.1. Basic Model

A two-compartment model with first-order absorption and elimination was sufficient to characterize vancomycin pharmacokinetics for CVVH patients. The inter-individual variability on clearance (CL) was added and described by the exponential model. The residual variability model was best described by a proportional error model.

#### 2.2.2. Covariate Model

As continuous variables were not normally distributed, Kendall’s correlation coefficient test was applied in the analysis. The correlation analysis showed no correlation between most of covariates. Sex was correlated with weight (*p* = 0.015), and weight was retained because it was more clinically significant. Weight was also correlated with ultrafiltration rate (correlation coefficient = −0.534, *p* = 0.027). However, ultrafiltration rate was a significant covariate to characterize in vitro removal, and their correlation was moderate, so both were retained.

The stepwise covariate modelling procedure (Supplementary Materials Tables S1–S3) resulted in the final model containing albumin level (ΔOFV = −9.115) and ultrafiltration rate (ΔOFV = −61.121) as significant covariates for CL. In addition, inclusion of allometrically scaled body weight on CL and volume of distribution (V) explained some variability and was retained in the final model. 

**Figure 1:**
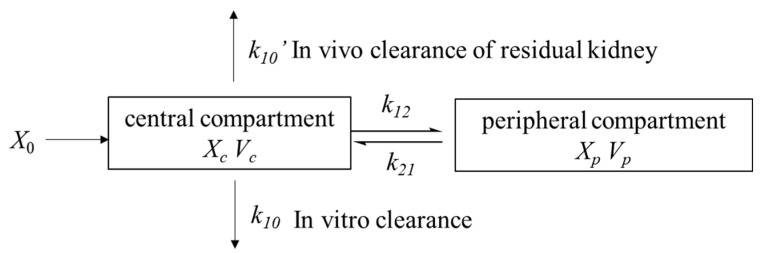
Two compartment model of vancomycin for critically ill patients undergoing CVVH.

The final model was therefore developed to describe the concentration-time profile of vancomycin (Equations (1)–(4)). The population estimates are summarized in Table 2. The structure diagram of final model was shown in Figure 1.

(1) $$CL=CL_{POP}\times {\left(WT/70\right)}^{0.75}\times {\left(29/ALB\right)}^{\alpha }+UFR\times \beta$$

(2) $$V_{c}=V_{c,POP}\times (WT/70)$$

(3) $$V_{p}=V_{p,POP}$$

(4) $$Q=Q_{POP}$$

#### 2.2.3. Model Evaluation

**Figure 2:**
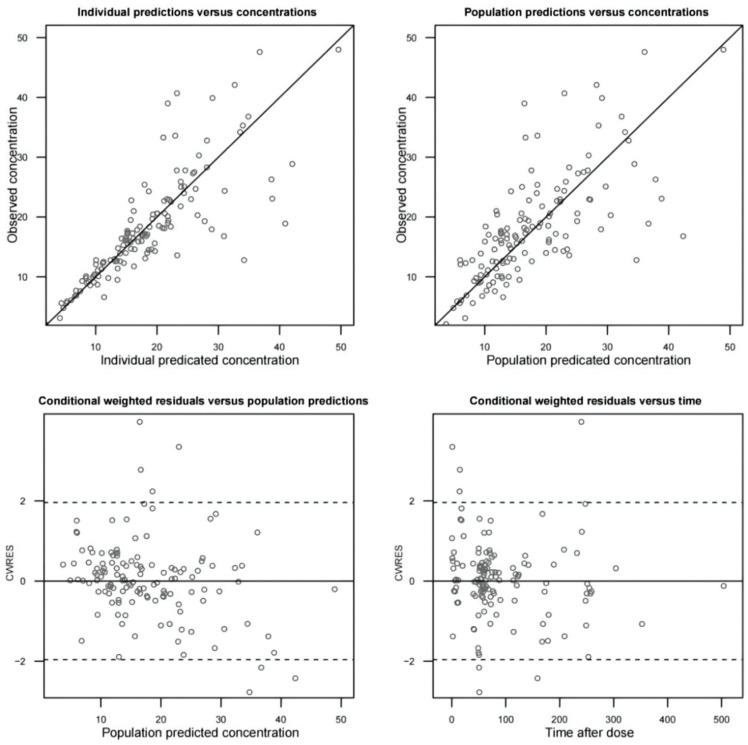
The typical goodness-of-fit diagnostic plots of the final model were as follows: individual prediction concentrations (IPRED) versus observed vancomycin plasma concentrations (DV) (**top left**), population prediction concentrations (PRED) versus observed vancomycin plasma concentrations (**top right**), conditional weighted residuals (CWRES) versus population prediction (PRED) concentrations (**bottom left**), and conditional weighted residuals versus time after dose (TIME) (**bottom right**). The diagonal lines in the upper panels represent lines of unity.

The typical goodness-of-fit (GOF) diagnostic plots are shown in Figure 2. The population prediction concentrations (PRED) and individual prediction concentrations (IPRED) based on the final model corresponded well with the observed concentrations and they were evenly distributed on both sides of the reference line (Y = X). The conditional weighted residuals (CWRES) values were distributed in the range of ±2, indicating the model fitted well.

The numerical predictive check (NPC) results are shown in Table 3. Taking the 90% prediction interval as an example, seven observations (5.34%) were below the 5th percentile and within the corresponding 95% confidence interval (0.76–12.98%). Nine observations (6.87%) were above the 95th percentile and within their 95% confidence interval (0.00–12.98%). In total, 12.21% of the observations were outside the 90% prediction range, which was close to the expected value of 10%, indicating that the prediction performance of the model was well.

### 2.3. Population PK/PD Analysis

**Figure 3:**
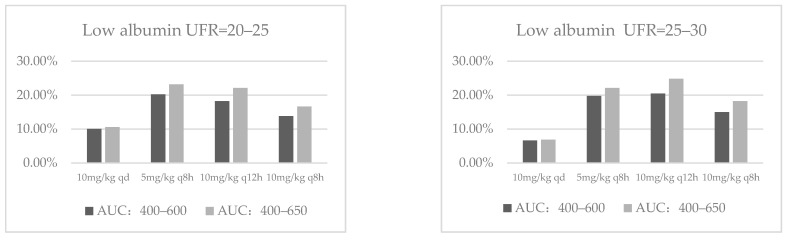

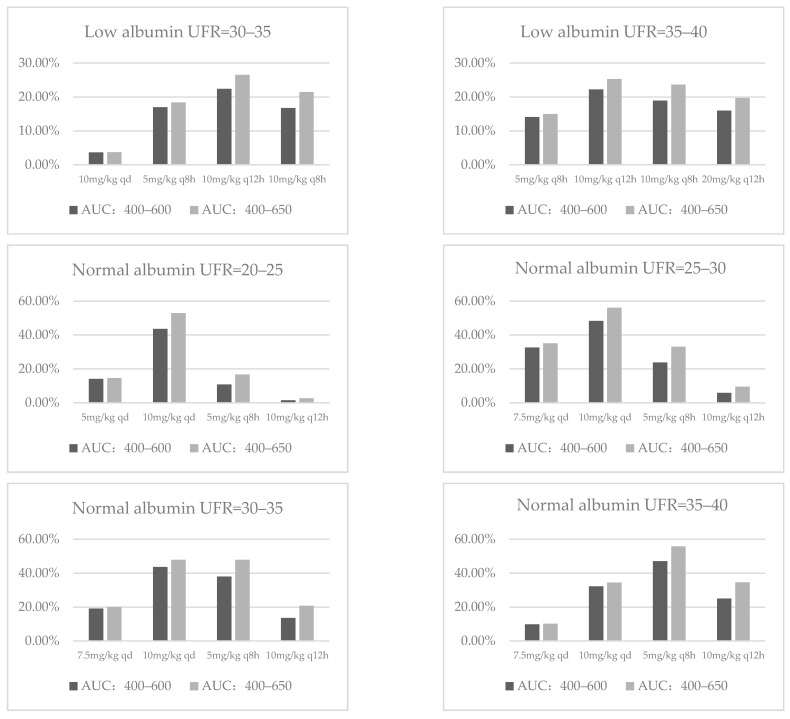
PTA for different dosage regimens in each subgroup.

Based on the covariates included in the model, the simulated population was classified according to the albumin level and the ultrafiltration rate. The albumin level was subdivided into low albumin (15–34 g/L) and normal albumin (35–55 g/L). Regarding the ultrafiltration rate, it was subdivided into four categories: 20–24.9 mL/kg/h, 25–29.9 mL/kg/h, 30–34.9 mL/kg/h and 35–40 mL/kg/h, according to the current published literatures and clinical practice. Figure 3 shows the probability of target attainment (PTA) for different dosage regimens in each subgroup.

In patients with normal albumin and UFR between 20 and 35 mL/kg/h, the recommended dosage regimen was 10 mg/kg qd. When UFR was between 35 and 40 mL/kg/h, the recommended dosage regimen was 5 mg/kg q8h. In patients with hypoalbuminemia and UFR between 20 and 25 mL/kg/h, the recommended dosage regimen was 5 mg/kg q8h. When UFR was between 25 and 40 mL/kg/h, the recommended dosage regimen was 10 mg/kg q12h.

## 3. Discussion

In this study, a prospective population PK analysis of vancomycin in 11 CVVH patients with 131 concentrations was performed. A two-compartment model with first-order elimination best described the data, as previously shown [24]. Ultrafiltration rate and albumin level significantly affected CL. The population typical vancomycin CL is 1.15 L/h, and there is an increase of 0.0377 L/h as the ultrafiltration rate increases by 1 mL/(kg∙h). The American revised consensus guideline of the therapeutic monitoring of vancomycin for serious methicillin-resistant *Staphylococcus aureus* infections [20] mentioned that the clearance of vancomycin by CVVH mainly depends on the ultrafiltration rate [25]. As we all know, only free drugs can be filtered out, drugs bound to plasma proteins have a larger molecular weight and have difficulty penetrating the filtration membrane. Albumin levels can affect the concentration of free drugs, so the clearance of a patient is correlated negatively with the albumin level. Here we only focus on the measured albumin levels, and it does not matter whether the patients received albumin supplementation. In this study, patients were divided into different subgroups based on ultrafiltration rate and albumin levels, and the initial dose regimen of vancomycin in different subgroups were recommended using Monte Carlo Simulation.

Several other studies investigated vancomycin initial dosage regimens for this special population, but the findings were inconsistent, and few studies implemented a population pharmacokinetic analysis. Most studies only considered the effect of ultrafiltration rate. In addition, some studies even directly recommend dosage regimen without considering covariates effect. A review of recommendations for antimicrobial dosing optimization during continuous renal replacement therapy [21] recommended 400–650 mg q12h for patients whose ultrafiltration rate are between 30 and 40 mL/kg/h. This regimen does not take into account of patients’ albumin level and weight. It was based on an original study [22] including ten patients, and seven of them had albumin levels below 35 g/L. Our study recommends 10 mg/kg q12h for patients with lower albumin at the same ultrafiltration rates range, and the two regimens are the same for 50–60 kg patients.

The American revised consensus guideline of the therapeutic monitoring of vancomycin for serious methicillin-resistant *Staphylococcus aureus* infections [20] recommended 7.5–10 mg/kg q12h when the ultrafiltration rate is between 20 and 25 mL/kg/h, but the regimen also does not take into account of patients’ albumin level. This regimen was based on an original study [23] included seven patients, seven of them with albumin levels below 35 g/L and only one patient with albumin levels within the normal range of 35 g/L to 55 g/L. Our study recommends 5 mg/kg q8h for patients with lower albumin and the same ultrafiltration rates range, and the daily dose is 15 mg/kg, which is the lower limit of 7.5–10 mg/kg q12h. The reason may be the different selected PK/PD targets. The previous study selected trough concentration 15–20 mg/L, whereas we chose AUC 400–650 mg∙h/L.

Other studies recommended lower doses than ours. An initial dosage regimen of 7.5–10 mg/kg every 12 h was previously recommended in CVVH patients [23]. In the same patients, the Sanford Guideline for Antimicrobial Therapy (2018) recommended 500 mg q24h–q48h for CVVH patients. The two regimens did not consider the ultrafiltration rates and albumin level. We recommend 10 mg/kg qd when the ultrafiltration rate is between 20 and 25 mL/kg/h and plasma albumin level is normal, consistently with the upper limit of the abovementioned regimens

It can be seen that current recommendations for initial dosage regimens of vancomycin in critically ill patients undergoing CVVH are not clear. The reasons may be that the variability in CVVH prescription was not unexpected [26], and many factors can affect vancomycin concentrations, such as the parameters of CVVH, baseline characteristics of the included patients and different PK/PD targets. Therefore, it is recommended choosing regimens similar to the clinical practice setting and conducting dose adjustment empirically. In addition, it is important to do therapeutic drug monitoring closely and make dose adjustments as necessary.

This study has several strengths. Firstly, we prospectively collected blood samples, which allows the model results to be more accurate than retrospective studies. Secondly, we recommended dosage regimens for different subgroups classified by albumin level and ultrafiltration rate, which has stronger clinical applicability. Thirdly, the PK/PD targets used in this study were the AUC between 400–600 and 400–650 mg∙h/L. Current evidence [20,27] shows that AUC better reflects the effectiveness and safety of vancomycin than trough concentration. Fourthly, compared with traditional pharmacokinetic analysis, covariate screening used in population pharmacokinetic analysis was helpful to find the factors affecting the pharmacokinetic characteristics of this special population, and PK/PD analysis could be used to recommend appropriate initial dosage regimens for different subgroups. In addition, the established PPK model combining the result of TDM can be used to accurately adjust the dosage of vancomycin and realize individualized drug administration.

The limitations of this study have also to be mentioned. Firstly, external validation was not performed, and conclusions are only applicable to similar clinical scenarios. Our study required that patients must receive CVVH treatment for at least 16 h daily, which may restrict extrapolation. Besides, the residual renal function was not included as a significant covariate, as most of the patients had the similar impaired renal function. Therefore, the extrapolation to patients with normal or nearly normal renal function was relatively poor because most of the patients were oliguric or anuric. Thirdly, the sample size was small and large samples are needed to validate our findings.

## 4. Materials and Methods

### 4.1. Patients

A single center prospective clinical trial was conducted from January 2018 to January 2020 at Peking University Third Hospital. Patients receiving vancomycin and CVVH simultaneously in the Intensive Care Unit were enrolled. Inclusion criteria were: (i) aged over 16 years old; (ii) patients with documented or suspected multidrug-resistant Gram-positive pathogens who were receiving intravenous vancomycin; (iii) CVVH treatment time equal to or longer than 72 h; (iv) CVVH daily treatment time equal to or longer than 16 h. Exclusion criteria were: (i) patients with hematologic diseases; (ii) pregnant women; (iii) patients whose estimated life expectancy was less than 48 h; (iv) patients who cannot obtain written informed consent. Intravenous vancomycin was routinely administered at empirical dose by doctors. The dosage regimens of patients included in this study were 0.5g qd/q12h/q8h, which were adjusted based the recommendation of Sanford Guide [28]. This study was approved by the Peking University Third Hospital Ethics Committee (reference number M2017114), and written informed consent was obtained from the authorized person of each participant.

### 4.2. Blood Sampling and Analytical Assay

Blood samples (1 mL) were collected at the baseline (before administration), 0.5 h, 1 h, 2 h, 4 h, 6 h, 8 h, 10 h and 12 h after the first dose of vancomycin when the patient had started CVVH treatment. We repeated the blood sampling at the third day. The specific time of blood collection may be fine-tuned according to the actual clinical practice, and the time of blood collection and drug administration should be accurately recorded. Collected blood samples were then centrifuged at 3000 rpm and sent to the Department of Clinical Laboratory for determination. Blood concentration of vancomycin was determined by commercial chemiluminescent microparticle immunoassay (CMIA) assay using the ARCHITECT platform with the ARCHITECT iVancomycin assay obtained from Abbott Laboratories Trading Co., Ltd. (Shanghai, China) [29,30]. The measurements were recorded to establish vancomycin population pharmacokinetic model.

### 4.3. Data Collection

Demographic data (gender, age, height, weight, and ethnicity), disease information (anamnesis, diagnosis, complications, APACHE II score and SOFA score [31]), vital signs (systolic blood pressure, diastolic blood pressure, heart rate, respiratory rate and temperature), urine volume, blood routine examination and blood biochemical parameters (red blood cell count, white blood cell count, platelet count, hemoglobin, alkaline phosphatase, aspartate aminotransferase, alanine aminotransferase, total bilirubin, total protein, albumin, blood urea nitrogen and serum creatinine), vancomycin use related information (dosage regimen and infusion rate) and CVVH parameters (blood flow rate, the diluting modes and ultrafiltration rate) were collected during the study.

### 4.4. Statistical Analysis

Descriptive statistics were performed to describe all variables. Discrete variables were expressed as counts and percentages. Continuous variables were expressed as means and standard deviation (SD) or medians and interquartile range (IQR), depending on the normality of their distribution (ShapiroWilk’s test) [4]. Pearson’s correlation coefficient or Kendall’s correlation coefficient was calculated for the continuous variables using SPSS 24.0 according to whether they were normally distributed. *T* test was used to judge the correlation between discrete variables and continuous variables. Covariates without correlation were screened for covariate model development.

### 4.5. Population PK Model Development

#### 4.5.1. Structural Model

The concentration–time profile was analyzed using a non-linear mixed-effects population approach with NONMEM (version 7.3.0; ICON Development Solutions, Ellicott City, MD, USA). Model building was assisted by Perl-speaks-NONMEM (PsN, version 4.7.0) and the graphical evaluation was performed with Microsoft Excel 2016, R (version 3.6.0) and Xpose (version 4.5.3).

The first-order conditional estimation method with interaction was applied to all model runs. One- and two-compartment models with linear elimination were compared to evaluate the best basic structural model. The model with the minimum Akaike information criterion (AIC) was selected as the optimal structure model. The typical population values of CL and V were estimated.

#### 4.5.2. Statistical Model

Inter-individual variability in vancomycin PK parameters was described by exponential error models: P_ij_ = P_pop_ × exp(η_ij_), where P_ij_ is the jth estimated pharmacokinetic parameter of the *i*th individual, P_pop_ represents the population typical value of the *j*th parameter and η_ij_ is a random variable distributed with a mean of zero and a variance of ω^2^. Residual variability was evaluated by comparing the following models (Equations (5)–(8)) [32]:


Additive error model: C_obs_ = C_pred_ + ε (5)

Proportional error model: C_obs_ = C_pred_ × (1 + ε) (6)

Combined error model: C_obs_ = C_pred_ × (1 + ε) + ε’ (7)

Exponential error model: C_obs_ = C_pred_ × exp(ε) (8)

In the above equations, C_obs_ and C_pred_ are the observed and predicted concentrations, and ε and ε’ are random variables distributed with a mean of zero and variances of σ^2^ and σ’^2^, respectively.

#### 4.5.3. Covariate Model

Influential covariates were explored using forward inclusion, followed by backward elimination procedure. Continuous covariates were normalized using population mean values. A reduction in objective function value (OFV, computed as −2 times the log-likelihood) of >3.84 (*p* < 0.05) was considered statistically significant for the inclusion of one additional parameter in the forward inclusion step. An increase in OFV of >10.83 (*p* < 0.001) was considered statistically significant in the backward elimination step [33]. Covariates were included in the final model if the following criteria were met: (i) the OFV was minimized and the goodness-of-fit was improved, (ii) clinical plausibility existed for incorporating the covariates; and (iii) the 95% CIs for the parameter estimates did not include zero [34].

#### 4.5.4. Model Evaluation

The goodness of fitting was examined by plotting GOF (individual prediction concentrations (IPRED) and population prediction concentrations (PRED) versus observed concentrations, respectively; PRED and time after dose versus conditional weighted residuals, respectively). A 2000-times resampling bootstrap approach was applied to evaluate the robustness of the final model. The results of the bootstrap analysis (median, 95% CI) were compared with the estimated values of the final model. Numerical predictive check (NPC) was used to assess the predictive performance of the final model (with 1000 simulations).

### 4.6. Population PK/PD Analysis

Monte Carol Simulation was conducted in different subgroups based on the covariates included in the final model. Assuming a minimal inhibitory concentration (MIC) of 1 mg/L, the 24-h area under the curve (AUC_24_) values of vancomycin should be maintained between 400 and 600 mg·h/L or between 400 and 650 mg·h/L to maximize efficacy and minimize the likelihood of nephrotoxicity [20,27]. A 10,000-patients simulation was performed using NONMEM and the probability of target attainment (PTA) was calculated for different dosage regimens. PTA was defined as the number of qualified patients divided by the number of simulation patients. The dosage regimen with the highest PTA was chosen as the final recommended one.

## 5. Conclusions

A two-compartment model with first-order elimination was sufficient to characterize vancomycin pharmacokinetics for CVVH patients. Albumin level and ultrafiltration rate can significantly affect CL. We recommend choosing the optimal initial vancomycin dosage regimens according to these two covariates. Future prospective studies with larger samples are needed to further validate the optimal dosage regimen of vancomycin for critically ill patients undergoing CVVH.

## Figures and Tables

**Table 1:** Demographic and clinical characteristics of 11 patients.

Characteristic	Value
Number of patients	11
Number of samples	131
Age, median [IQR] (range), y	63 [30.5] (20–83)
Body weight, median [IQR] (range), kg	70 [22.5] (52–90)
Urinary output, median [IQR] (range), mL	300 [436.8] (0–3340)
Creatinine clearance before CVVH, median [IQR] (range), mL/min	29.1 [13.6] (11–66)
Albumin, median [IQR] (range), g/L	28.6 [5.1] (26–39)
Ultrafiltration rate, median [IQR] (range), mL/kg/h	33.3 [6.5] (18–39)
APACHE II score, median [IQR] (range)	24 [1] (9–27)
SOFA score, median [IQR] (range)	9 [3] (5–12)
SEX, no. (%)	
Female	5 (45.5%)
Male	6 (54.5%)

**Table 2:** Population estimates from the final model and bootstrap analysis results.

Parameters	Values (%RSE)	Bootstrap	
		Median	95%CI
CLPOP (L/h)	1.15 (17)	1.17	0.63–1.69
Vc (L)	16.9 (11)	16.77	11.78–23.35
Vp (L)	25.9 (19)	26.72	15.38–38.74
Q (L/h)	7.72 (18)	7.65	4.60–21.42
α,ALB effect on CL	5.52 (20)	5.00	1.38–7.89
β,UFR effect on CL	0.0377 (14)	0.0378	0.024–0.056
IIV CL	0.0647 (28)	0.056	0.0126–0.101
RV (proportional)	0.0507 (22)	0.0466	0.023–0.068

CL = clearance; subscript POP = population typical value; V_c_ = volume of distribution of the central compartment; V_p_ = volume of distribution of the peripheral compartment; Q = intercompartment clearance; IIV = interindividual variability; RV = residual variability; %RSE = percent relative standard error of the estimate, calculated as SE/parameter estimate × 100 (for variability terms, this is the %RSE of the variance estimate).

**Table 3:** NPC results.

	Points below PI (Count)	Points below PI (%)	95% CI below (%)	Points above PI (Count)	Points above PI (%)	95% CI above (%)
0% PI	54	41.22	32.06–67.18	77	58.78	32.82–67.94
20% PI	42	32.06	23.66–56.49	54	41.22	23.66–57.25
40% PI	36	27.48	15.26–45.80	40	30.53	15.27–46.56
50% PI	28	21.37	11.45–41.22	34	25.95	10.68–41.22
60% PI	24	18.32	8.40–34.35	27	20.61	7.63–35.11
80% PI	14	10.69	2.29–21.37	16	12.21	2.29–21.37
90% PI	7	5.34	0.76–12.98	9	6.87	0.00–12.98
95% PI	3	2.29	0.00–7.63	6	4.58	0.00–8.40

PI: prediction interval; CI: confidence interval.

## Data Availability

The data presented in this survey are available on reasonable request through the corresponding author. The data are not publicly available due to privacy restrictions.

## Supplementary Materials

The following are available online at www.mdpi.com/article/10.3390/antibiotics10111392/s1, Table S1: The first round of forward inclusion results. Table S2: The second round of forward inclusion results. Table S3: The results of backward elimination.
