# Supplementary material for: Dose Optimization of Vancomycin for Critically Ill Patients Undergoing CVVH: A Prospective Population PK/PD Analysis
Source: Antibiotics (Basel). 2021 Nov 13;10(11):1392. doi: 10.3390/antibiotics10111392 (PMC8614878; doi:10.3390/antibiotics10111392)
Supplement: Supplementary file 1 [file antibiotics-10-01392-s001.zip › antibiotics-1419488-supplementary.pdf]

Supplementary Materials

Table S1. The first round of forward inclusion results.

No. of model	Description	Function form	OFV	ΔOFV	*P* value
1	base model	-	585.337	0	-
2	add AGE on CL	power	612.126	26.789	> 0.05
3	add SEX on CL	proportional	609.596	24.259	> 0.05
4	add WT on CL	power	611.876	26.539	> 0.05
5	add BMI on CL	power	608.899	23.562	> 0.05
6	add APA on CL	power	612.288	26.951	> 0.05
7	add SOFA on CL	power	610.306	24.969	> 0.05
8	add SBP on CL	power	606.092	20.755	> 0.05
9	add DBP on CL	power	607.909	22.572	> 0.05
10	add HR on CL	power	597.57	12.233	> 0.05
11	add RE on CL	power	609.334	23.997	> 0.05
12	add TEM on CL	power	612.162	26.825	> 0.05
13	add UV on CL	exponential	606.985	21.648	> 0.05
14	add PLT on CL	linear	601.857	16.52	> 0.05
15	add RBC on CL	exponential	611.71	26.373	> 0.05
16	add WBC on CL	power	612.274	26.937	> 0.05
17	add ALP on CL	power	611.234	25.897	> 0.05
18	add ALT on CL	exponential	611.903	26.566	> 0.05
19	add TBIL on CL	power	606.065	20.728	> 0.05
20	add ALB on CL	linear	579.983	-5.354	< 0.05*
21	add BUN on CL	power	602.017	16.68	> 0.05
22	add CLCR on CL	exponential	604.892	19.555	> 0.05
23	add UFR on CL	linear	527.977	-57.36	< 0.05*

AGE, age; SEX, gender; WT, weight; BMI, body mass index; APA, Acute Physiology and Chronic Health Evaluation II (APACHE II) score; SOFA, Sequential Organ Failure Assessment; SBP, systolic blood pressure; DBP, diastolic blood pressure; HR, heart rate; RE, respiratory rate; TEM, temperature; UV, urine volume; PLT, platelet count; RBC, red blood cell count; WBC, white blood cell count; ALP, alkaline phosphatase; ALT, alanine aminotransferase; TBIL, total bilirubin; ALB, albumin; BUN, blood urea nitrogen; CLCR, creatinine clearance; UFR, ultrafiltration rate.

*The covariate was statistical significant.

Table S2. The second round of forward inclusion results.

No. of model	Description	Function form	OFV	ΔOFV	*P* value
24	add ALB on CL based on 23	power	509.525	-18.452	< 0.05*

ALB, albumin.

*The covariate was statistical significant.

Table S3. The results of backward elimination.

No. of model	Description	Function form	OFV	ΔOFV	*P* value
25	eliminate ALB based on 24	linear	527.977	18.425	< 0.001*
26	eliminate UFR based on 24	power	578.111	68.586	< 0.001*

ALB, albumin; UFR, ultrafiltration rate.

*The covariate was statistical significant.
